# Unmet patient needs in monocarboxylate transporter 8 (MCT8) deficiency: a review

**DOI:** 10.3389/fped.2024.1444919

**Published:** 2024-07-22

**Authors:** Andrew J. Bauer, Bethany Auble, Amy L. Clark, Tina Y. Hu, Amber Isaza, Kyle P. McNerney, Daniel L. Metzger, Lindsey Nicol, Samuel R. Pierce, Richard Sidlow

**Affiliations:** ^1^ The Thyroid Center, Division of Endocrinology and Diabetes, The Children’s Hospital of Philadelphia, Philadelphia, PA, United States; ^2^ Department of Pediatrics, Perelman School of Medicine at the University of Pennsylvania, Philadelphia, PA, United States; ^3^ Medical College of Wisconsin, Children’s Wisconsin, Milwaukee, WI, United States; ^4^ Department of Pediatrics, Saint Louis University School of Medicine, St. Louis, MO, United States; ^5^ Department of Pediatric Endocrinology and Diabetes, SSM Health Cardinal Glennon, St. Louis, MO, United States; ^6^ Department of Pediatrics, Division of Endocrinology, University of California San Francisco, San Francisco, CA, United States; ^7^ Diabetes Education Program, Washington University in St. Louis, St. Louis, MO, United States; ^8^ The Endocrinology & Diabetes Unit, British Columbia Children’s Hospital, Vancouver, BC, Canada; ^9^ Department of Pediatrics, University of British Columbia, Vancouver, BC, Canada; ^10^ Department of Pediatric Endocrinology, Oregon Health & Science University Doernbecher Children’s Hospital, Portland, OR, United States; ^11^ Division of Endocrinology, Oregon Health & Science University, Portland, OR, United States; ^12^ Division of Rehabilitation Medicine, The Children’s Hospital of Philadelphia, Philadelphia, PA, United States; ^13^ Department of Medical Genetics and Metabolism, Valley Children’s Hospital, Madera, CA, United States

**Keywords:** MCT8 deficiency, MCT8, Allan-Herndon-Dudley syndrome, AHDS, developmental delay, T3, rare diseases, thyroid hormone

## Abstract

Monocarboxylate transporter 8 (MCT8) deficiency is a rare, X-linked disorder arising from mutations in the *SLC16A2* gene and resulting from dysfunctional thyroid hormone transport. This disorder is characterized by profound neurodevelopmental delay and motor disability due to a lack of thyroid hormone in the brain, and coexisting endocrinological symptoms, due to chronic thyrotoxicosis, resulting from elevated thyroid hormone outside the central nervous system (CNS). In February 2024, we reviewed the published literature to identify relevant articles reporting on the current unmet needs of patients with MCT8 deficiency. There are several main challenges in the diagnosis and treatment of MCT8 deficiency, with decreased awareness and recognition of MCT8 deficiency among healthcare professionals (HCPs) associated with misdiagnosis and delays in diagnosis. Diagnostic delay may also be attributed to other factors, including the complex symptomology of MCT8 deficiency only becoming apparent several months after birth and pathognomonic serum triiodothyronine (T3) testing not being routinely performed. For patients with MCT8 deficiency, multidisciplinary team care is vital to optimize the support provided to patients and their caregivers. Although there are currently no approved treatments specifically for MCT8 deficiency, earlier identification and diagnosis of this disorder enables earlier access to supportive care and developing treatments focused on improving outcomes and quality of life for both patients and caregivers.

## Introduction

Monocarboxylate transporter 8 (MCT8) deficiency—also known as Allan-Herndon-Dudley Syndrome (AHDS)—is a rare, X-linked genetic disorder that has a profound effect on the lives of patients and their caregivers (1–4). This disorder is associated with neurodevelopmental disabilities, hypermetabolic malnutrition, and tachycardia, with one in three affected individuals dying in childhood (1, 5, 6). MCT8 deficiency arises from a pathogenic mutation in the *SLC16A2* gene located on the X-chromosome, which encodes MCT8, a thyroid hormone transporter protein widely expressed in human tissues (1, 2, 7, 8). MCT8 is instrumental in the cellular uptake of the inactive prohormone thyroxine (T4) and the bioactive triiodothyronine (T3), where T3 is critical to several important physiological processes (7, 9–11). Pathogenic variants in the *SLC16A2* gene cause reduced or absent thyroid hormone uptake into the brain, resulting in altered neural development and myelination (1, 9–11). However, because thyroid hormone can enter cells in the rest of the body independent of MCT8, affected individuals concomitantly present with signs and symptoms of peripheral hyperthyroidism, including tachycardia and altered metabolism (1, 2, 5–7, 9, 12–14). As an X-linked disorder, the condition predominantly affects males, although a few confirmed cases have been reported in females (1, 5, 15). The estimated prevalence based on diagnosed cases to date is fewer than one in a million (16).

**Figure 1:**
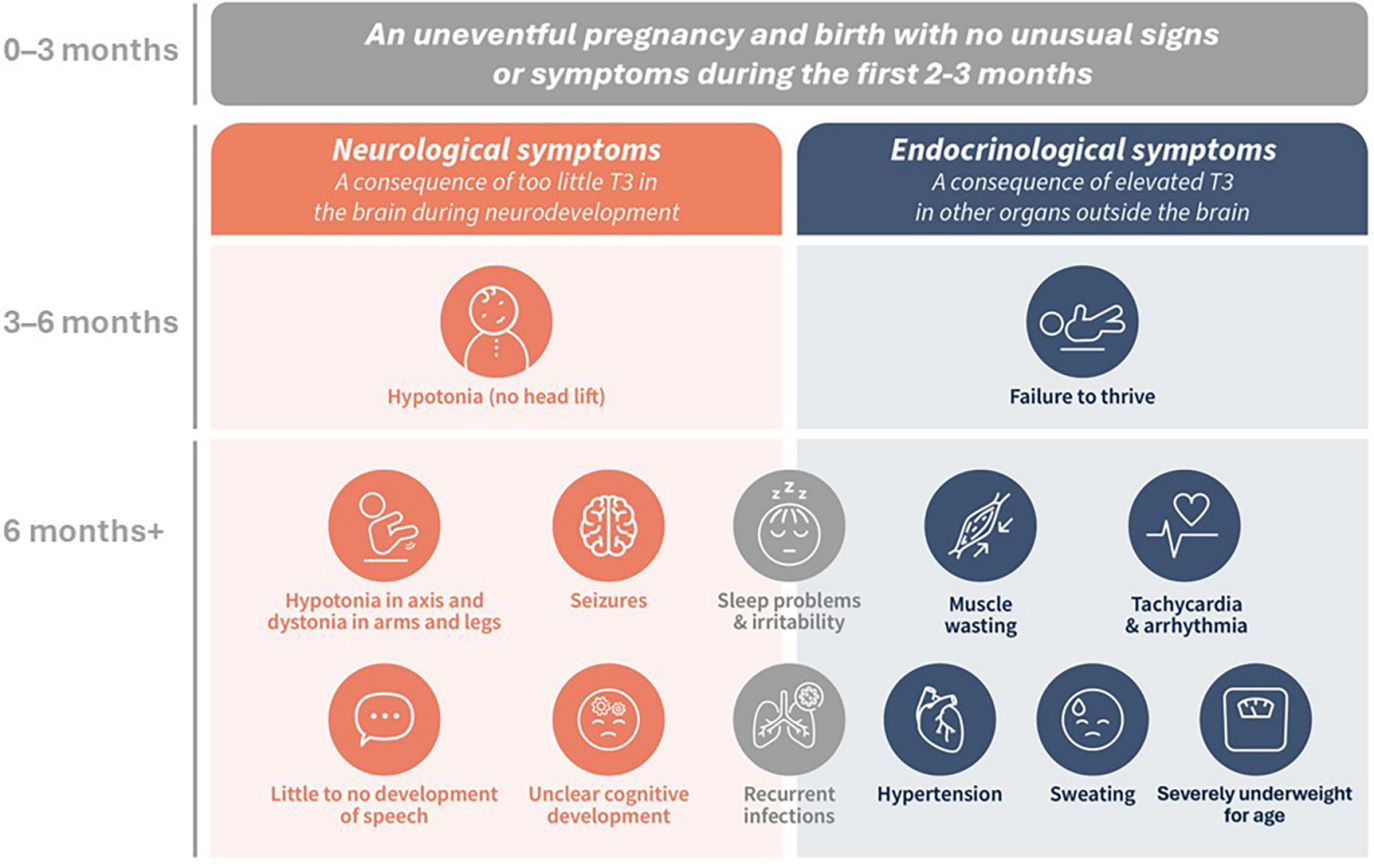
The co-existing neurological and endocrinological symptoms indicative of MCT8 deficiency. (1, 2, 5, 6, 8, 13, 14).

Symptoms of this disorder usually present after the first 2–3 months of life, characterized by hypotonia (lack of head lift), developmental delay, and failure to thrive (Figure 1). Patients may initially be referred to a pediatric neurologist, pediatric endocrinologist, or pediatric gastroenterologist, but over time, their symptoms become progressively severe and include cognitive issues, gross and fine motor delay, movement disorders such as hypokinesia and dystonia, mixed hypotonia with axial spasticity, limited ability to communicate, lack of weight gain, lack of increase in muscle tone, sleep problems, thyroid hormone abnormalities, and cardiac complications, alongside the presenting symptoms (1, 2, 4–6, 8, 13, 14, 17, 18). Once a diagnosis is established, a multidisciplinary team is best suited to care for the patient (4, 5). This team can include a primary care provider (PCP; general pediatrician or family physician), a pediatric neurologist, physical, occupational, speech, and feeding specialists, a pediatric gastroenterologist with a dietician, geneticists and genetic counselors, and a family social worker and/or case manager to help families and caregivers navigate the daily, complex, life-impacting care needs of their child (1, 4, 5).

Currently, formal guidelines for the diagnosis and management of MCT8 deficiency do not exist, with diagnosis based on clinical signs and symptoms with a characteristic pattern of thyroid hormone levels in the blood, and confirmatory genetic testing (1). Current treatment options are limited to supportive and symptomatic therapies. Preliminary data suggest the importance of an early diagnosis to optimize the potential benefit of treatment options currently under development that target the complex underlying pathology of the disorder (5, 6, 16, 19).

In this review, we aim to highlight the current challenges in MCT8 deficiency diagnosis and management. We hope that raising awareness of MCT8 deficiency will aid in the earlier identification of the disorder and enable prompt intervention and access to appropriate care, helping to improve the quality of life of the patients and their caregivers.

## Methods

In February 2024, the published literature was reviewed to identify relevant articles reporting on the evaluation and management of patients with MCT8 deficiency. We included publications from PubMed limited to the past five years and only those published in English. Older publications were used where appropriate for introductory or background information. The initial search term string used in PubMed was: AHDS OR Allan-Herndon-Dudley syndrome OR MCT8 deficiency OR MCT8. Subsequent searches added the following additional terms to confirm relevant articles had not been missed in the initial search: T3, T4, rare, developmental delay, newborn screening, pediatric, paediatric, resistance to thyroid hormone, dystonia, hypotonia, hypokinesia, leukoencephalopathy, and failure to thrive. A search in PubMed using the following MeSH terms did not identify additional publications to the first search string: MCT8[All Fields] AND [“deficiency"(Subheading) OR deficiency(Text Word)] AND “Allan-Herndon-Dudley syndrome” [Supplementary Concept].

## Results

### Pathogenesis of MCT8 deficiency

MCT8 is the only thyroid hormone transporter involved in the transfer of T3 and T4 into and out of the human brain, and its dysfunction results in reduced levels of T3 and T4 in the central nervous system (CNS) (10, 20). Outside the CNS, other thyroid hormone transporters are involved in the movement of T3 and T4 into and out of cells, although with less specificity (21). Loss of MCT8 function is thought to affect thyroid hormone sensitivity in the hypothalamus and pituitary gland, decrease T4 secretion from the thyroid, and increase T4 “trapping” in the kidneys, a combination thought to contribute to elevated serum T3 levels (20). Therefore, MCT8 deficiency results in two co-existing sets of symptoms of combined but opposing thyroid hormone dysfunction: central hypothyroidism secondary to reduced T3 in the brain during critical periods of neurodevelopment, and peripheral hyperthyroidism secondary to elevated T3 in other organs and body systems (Figure 1) (1, 20). With most neurodevelopment occurring during gestation and through to three years of age, the goal is to diagnose patients as early as possible and develop treatments that could be used during this critical window to attenuate the severity of neurodevelopmental and neurocognitive deficits (1, 2, 5).

### There is a lack of knowledge concerning MCT8 deficiency

The first challenge in diagnosing patients with MCT8 deficiency is the lack of awareness and clinical pattern recognition of the disorder among healthcare professionals (HCPs). Pregnancy and the first few months of life tend to progress uneventfully, so there is no indication of MCT8 deficiency when these children are first seen by PCPs (1). In the months that follow, symptoms such as failure to thrive, global developmental delay, and hypotonia become more prominent but are still non-specific (1). By 6 months of age, when additional symptoms develop, e.g., dystonia and tachycardia, the differential diagnosis is still broad (1, 2, 5). Data from the International MCT8 Deficiency Registry—a prospective registry of parents and physicians caring for patients with MCT8 deficiency—showed that 58% (18/31) of parents reported knowledge of MCT8 deficiency among medical professionals they encountered to be “bad” or “very bad”, illuminating the need for increased awareness and education of HCPs (4).

### Diagnosis of MCT8 deficiency is often delayed

The lack of knowledge concerning the natural history, the phenotypic spectrum, and the pathognomonic thyroid function abnormalities of MCT8 deficiency all contribute to the delayed diagnosis of affected individuals (6). The reported median age of diagnosis is 24 months (IQR 12.0–60.0) (6). Some of this delay may be unavoidable due to MCT8 deficiency being asymptomatic until 2–3 months of age (Figure 1). However, the median time from the onset of symptoms to a diagnosis has been reported as 18 months (IQR 7.8–63.0), associated with a frustrating and stressful experience for families (6). Encouragingly, more recent data from the International MCT8 Deficiency Registry reported a shorter diagnostic delay in patients born in or after 2017 compared with patients born before 2017 (8 months vs. 19 months, respectively; *P* < 0.0001) (4).

**Figure 2:**
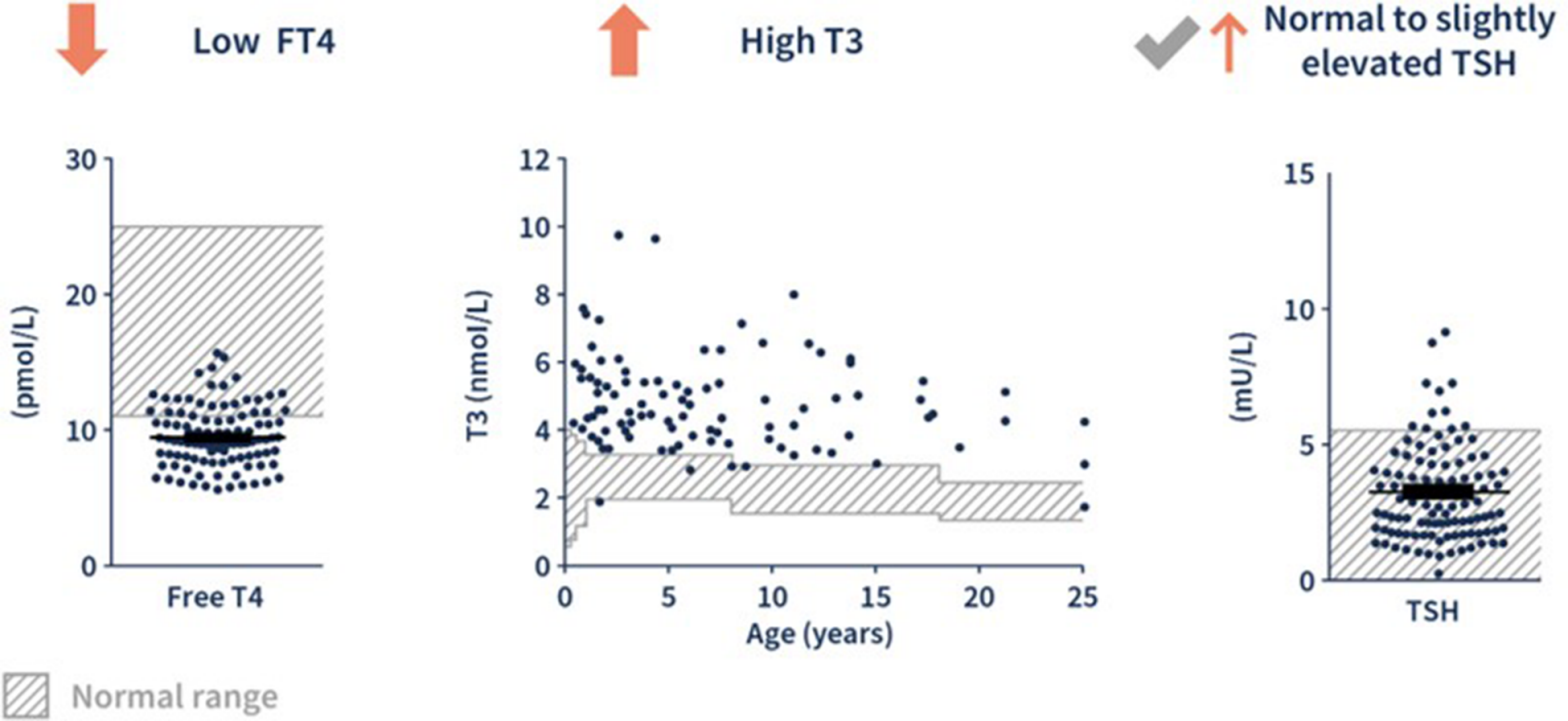
The characteristic pattern of thyroid hormone levels in MCT8 deficiency. (1, 6) Adapted from Groeneweg et al. 2020. (6) FT4, free T4.

The diagnosis of MCT8 deficiency is based on a combination of neurological and endocrinological clinical findings with a characteristic thyroid hormone profile: elevated T3 with low FT4 and normal or slightly elevated TSH (Figure 2). Movement disorders are a frequent feature of MCT8 deficiency (18). In a study of twenty-seven male patients with genetically confirmed MCT8 deficiency [mean age at evaluation: 9.3 years (range 0.9–18.5)], hypokinesia, often associated with hypomimia and global hypotonia, was present in twenty-five patients and was the predominant movement disorder in nineteen of those patients (18). Dystonia was also observed in twenty-five patients and the predominant movement disorder in a minority of cases (5/27). In eleven patients, exaggerated startle reactions and/or other paroxysmal non-epileptic events were observed (18). Phenotypic craniofacial features (including long narrow face, open mouth, tented lip, and ear abnormalities), brain imaging abnormalities (including delayed myelination), and cardiac arrhythmia or tachycardia, support a potential diagnosis of MCT8 deficiency (1, 5). A confirmed diagnosis of MCT8 deficiency follows the identification of a pathogenic mutation in the *SLC16A2* gene (1).

### Differential diagnosis and MCT8 deficiency

In individuals with MCT8 deficiency, the broad differential diagnoses include cerebral palsy, Pelizaeus-Merzbacher (like) disease, *MECP2* duplication syndrome, and mitochondrial disorders. Patients with these disorders also have developmental delay and hypotonia, with either an X-linked or autosomal recessive inheritance pattern, and many of these disorders are more common than MCT8 deficiency (1, 4, 14, 22–24).

### Diagnostic thyroid hormone testing for MCT8 deficiency

Current newborn screening (NBS) was established to help identify congenital hypothyroidism and does not include serum T3 levels (23–26). This screening is usually performed in the first few days after birth, and routinely in the first 24 h in the United States (US) (23, 25, 26). Infants born with MCT8 deficiency have normal TSH and T4 based on gestational age with the pathognomonic rise in T3 levels not occurring until approximately four months of age (6, 26). Therefore, simply including T3 in the NBS protocol would not distinguish MCT8 deficiency from other thyroid hormone disorders (6). The only characteristic MCT8-related thyroid hormone alteration that is present within the newborn period is a low reverse T3 (rT3), however, this test may not be readily available and is infrequently performed (26).

After four months of age, serum thyroid hormone levels have a characteristic “fingerprint” indicative of MCT8 deficiency as shown in Figure 2 (1, 2):

• Low normal to decreased free T4 (unbound to thyroid hormone transport proteins) or total T4 (bound and unbound)
• Elevated free or total T3
• Normal to slightly elevated thyroid stimulating hormone (TSH)

Outside of the newborn period, the most common obstacle to diagnosis is the infrequent assessment of serum T3 in the evaluation of a child with signs and symptoms consistent with MCT8 deficiency (1). In a review of the thyroid hormone laboratory values associated with other thyroid disorders, only resistance to thyroid hormone α (RTH-α) has a similar pattern to MCT8 deficiency (Table 1) (27, 28). Both RTH-α and MCT8 deficiency may be associated with neurocognitive deficits and developmental delay, however, RTH-α is associated with bradycardia and skeletal anomalies, while MCT8 deficiency is associated with tachycardia and failure to thrive (1, 5, 28). Thus, the measurement of T3 is vital in identifying the characteristic MCT8 deficiency thyroid hormone “fingerprint”. If T3 is elevated, confirmatory genetic testing should be performed (26).

**Table 1:** A summary of thyroid function disorders according to thyroid hormone levels. (26, 28) Adapted from Kağizmanli et al. 2023. (28) TSH, thyroid stimulating hormone; T4, thyroxine [as free T4 (FT4) or total T4 (TT4)]; T3, triiodothyronine [as free T3 (FT3) or total T3 (TT3)].

Thyroid function disorder	TSH	T4	T3
Euthyroid sick syndrome	LOW	NORMAL/LOW	LOW
Graves’ Disease	LOW	HIGH	HIGH
Neonatal thyrotoxicosis	LOW	HIGH	HIGH
Hashitoxicosis	LOW	HIGH	HIGH
Biotin interference	LOW	HIGH	HIGH
Central hypothyroidism	NORMAL/LOW	LOW	NORMAL/LOW
Resistance to thyroid hormone α	NORMAL	NORMAL/LOW	NORMAL/HIGH
Resistance to thyroid hormone β	NORMAL	HIGH	HIGH
MCT8 deficiency	NORMAL/HIGH	LOW	HIGH
Hashimoto's thyroiditis	HIGH	LOW	N/A
Congenital/acquired hypothyroidism	HIGH	NORMAL/LOW	NORMAL/LOW
Subclinical hypothyroidism	HIGH	NORMAL	NORMAL
TSHoma	HIGH	HIGH	HIGH

### Magnetic resonance imaging (MRI)

In addition to the characteristic thyroid hormone “fingerprint”, neurological evaluation with MRI to assess brain myelination may be informative (11). A lack of MCT8, and resultant T3 deprivation in the brain, negatively affects oligodendrocyte maturation and function (11). Brain MRIs in children under five years of age with MCT8 deficiency have shown diffusely abnormal white matter indicative of severely delayed myelination or hypomyelination, particularly in the deep anterior white matter (1, 11). Hypomyelination is reported to improve over time (1, 11) and this may be accelerated by developing treatments that can increase T3 concentration in the CNS (11, 29, 30).

### Genetic testing

The diagnosis of MCT8 deficiency is confirmed by the presence of a pathogenic mutation in the *SLC16A2* gene (1). The relationship between mutations in the gene, located at Xq13, and the presentation of MCT8 deficiency symptoms was first reported in 2004 (8, 31, 32). The relatively recent knowledge localizing the gene may mean that some adult males with unexplained neurodevelopmental or cognitive delays or impairment may have undiagnosed MCT8 deficiency (33). As an X-linked disorder, there is a 50% chance that male children will be affected if their mother is a carrier of a pathogenic *SLC16A2* mutation (5). The mutation may also develop *de novo* during fetal development through germline mosaicism (1, 5). Although most patients are male, a few heterozygous females have been described with clinical symptoms consistent with MCT8 deficiency resulting from chromosomal translocations and non-random X-inactivation; these individuals often have milder symptoms and less severe thyroid hormone test abnormalities (1, 5, 15, 34).

The strategy for genetic testing can be gene-targeted (single-gene testing or multigene panels, including custom-designed panels) or comprehensive (whole exome sequencing, exome array, or whole genome sequencing) (Table 2) (1). If a gene panel of comprehensive testing is ordered, it is important to verify that the sequencing and analysis of the *SLC16A2* gene is included.

**Table 2:** *SLC16A2* testing methodologies for MCT8 deficiency diagnosis. (1) HCP, healthcare professional.

Methodology	Utility
Gene-targeted sequencing	
Single gene	Known mutation testing in the family of a confirmed patient with MCT8 deficiency HCP is confident of MCT8 deficiency diagnosis from symptomology and other tests
Multigene	MCT8 deficiency diagnosis or differential diagnosis from custom arrays or commercially available panels for similar disorders
Comprehensive sequencing	
Whole exome	MCT8 deficiency diagnosis or differential diagnosis and copy number variant detection Prior knowledge of likely gene(s) involved is not required
Whole genome	MCT8 deficiency diagnosis or differential diagnosis and copy number variant detection, including in regulatory/intergenic regions Prior knowledge of likely gene(s) involved is not required
Exome array	MCT8 deficiency diagnosis by detection of multi-exon copy number variations

A wide spectrum of pathogenic *SLC16A2* mutations has been characterized to date with an increasing number of new variants, and previously described variants of unknown significance (VUS), regularly added to this list, based on an improved understanding of the clinical phenotype (13, 35–42). Therefore, sequencing test results that previously did not lead to a diagnosis should be periodically reanalyzed and updated to provide accurate diagnoses (43). Genetic counseling is recommended for all families in which a child is being tested for an *SLC16A2* mutation to explain the significance of an MCT8 deficiency diagnosis, to discuss and coordinate the testing of parents and siblings of an affected child, and to consider the risks and options for future pregnancies (1, 20).

### A multidisciplinary care team is necessary for patients with MCT8 deficiency

The need for multidisciplinary care is present from the start of the patient's journey, even before the diagnosis is confirmed. The initial encounter with the health care system is most commonly when the child presents to a PCP for routine wellness examinations during the first year of life (1). Children with signs of MCT8 deficiency, such as failure to thrive, tachycardia, hypotonia, and delayed developmental milestones, may then be referred to a developmental pediatrician, pediatric neurologist, cardiologist, endocrinologist, gastroenterologist, or nutrition specialist, depending on the individual patient's specific symptoms and severity of presentation (1, 4). One of these subspecialists may order thyroid function tests and/or refer the patient to a clinical pediatric genetics team for confirmatory *SLC16A2* testing (1). Once diagnosed, children and their caregivers will require assistance from multiple specialists for symptomatic treatment and surveillance. The addition of a social worker and/or case manager to the team is also critical to help the family navigate the health care system (1, 4).

In the recent International Prospective Registry study of patients with MCT8 deficiency, feeding problems were cited as a frequent concern, with difficulty in gaining weight reported as a general symptom (4). However, only 19% (6/32) of patients surveyed reported a pediatric gastroenterologist as part of their care team, and only 31% (11/36) of patients were provided with dietary advice from a dietician or nutrition expert (4). Only 12.5% (2/16) of patients who reported feeding problems had a feeding tube (4). Being severely underweight is a common symptom of MCT8 deficiency and is associated with a higher mortality risk compared with patients of normal weight for their age so optimizing nutritional care is vital (6, 10). In addition, children with MCT8 deficiency are at risk of aspiration pneumonia, so swallowing function should be regularly assessed via speech therapy and radiographic swallowing studies (1, 6). Sudden death, potentially related to cardiac arrhythmia, associated with thyrotoxicosis, and complicated by malnourishment and hypotonia, is a common cause of death in individuals with MCT8 deficiency (6). Despite the cardiac symptoms of this disorder, only 6% (2/32) of patients in the International Prospective Registry study were referred to a pediatric cardiologist (4). In addition, 1 in 5 affected children did not receive regular physical therapy, despite profound neuromuscular deficits (4).

Difficulties with sleeping are a commonly reported challenge, particularly in children under five years of age, and this negatively impacts the quality of life of patients and caregivers (4). Orthopedic specialists should be consulted to assess for hip dysplasia, which may cause discomfort, and patients should be evaluated for scoliosis, which may impede respiratory function and sleep in children with MCT8 deficiency (1). In a study investigating the preferred therapeutic goals of caregivers, all the participants surveyed (22/22; 100%) wished for improvement in development (motor, verbal, or social skills), particularly the achievement of gross motor milestones (12). Other goals listed include improved head control (59%) and sitting ability (50%), weight gain (36%), improvement of expressive language skills (32%), reduction of reflux (27%), gastric tube independence (18%), and a reduction of dystonia/spasticity (18%) and associated dysphagia (27%) (12).

The earlier a patient is diagnosed, the earlier an optimal multidisciplinary team of specialists can address the everyday challenges of caring for a patient with MCT8 deficiency (4). Despite potential improvements in recognition and time to diagnosis, clinical guidelines and the availability of an approved targeted treatment for MCT8 deficiency are needed (1). Current treatment relies on the use of supportive therapies to improve strength and mobility, delivered by a cohesive, multidisciplinary team and focused on helping affected individuals and their caregivers manage the day-to-day challenges of this disorder (4, 13, 20).

### Current therapies under investigation

There are no approved therapies for the treatment of MCT8 deficiency. Current thyroid hormone medications that are approved for use in other thyroid conditions are not targeted or effective in managing the dysregulation of thyroid hormone transport in MCT8 deficiency. The aim of managing MCT8 deficiency is to restore adequate thyroid hormone signaling to the brain and treat the peripheral thyrotoxic state caused by elevated serum T3 levels outside the brain (2). Treatment with carbimazole, methimazole (MMI), or propylthiouracil (PTU)—in combination with levothyroxine (LT4)—has been tried in an attempt to reduce T3 and replace thyroid hormone in a controlled manner (16, 19, 44). Unfortunately, this approach does not address the inability of thyroid hormone to access the brain, and to date, these medications have proven ineffective, while at the same time exposing patients to potentially serious side effects, including drug-induced neutropenia and hepatotoxicity (19, 44). In the US, PTU carries an FDA black box warning and is contraindicated for use in pediatric patients secondary to a risk of drug-induced liver failure in 1 in 2000–4000 children, which may be fatal or require liver transplantation (44). MMI is also associated with a risk of cholestatic hepatotoxicity, agranulocytosis, pancytopenia, and aplastic anemia, although these adverse reactions are not common (44).

### Therapeutic agents in development

The optimal therapy for MCT8 deficiency would alleviate both the neurological symptoms caused by decreased levels of T3 in the brain, as well as the endocrinological symptoms associated with elevated serum T3 levels in the rest of the body (19). Targeting the symptoms associated with thyrotoxicosis is more straightforward since thyroid hormone enters cells in tissues outside the brain via non-MCT8 dependent mechanisms, and lowering thyroid hormone in the serum leads to decreased signs and symptoms of hyperthyroidism (17). Targeting CNS hypothyroidism is more complicated as the opportunity to have an impact on brain development is time-dependent, starting during organogenesis (during the first trimester of pregnancy) with decreasing impact over the first three years of life, and a suitable drug needs to circumvent the defective MCT8 transporter, cross the blood-brain barrier and enter cells in the CNS (2, 5). It is also thought a lack of thyroid hormone in brain development and abnormal functioning of dopaminergic circuits of the basal ganglia may be related to the movement disorders frequently seen in MCT8 deficiency (18). Thyromimetics under research investigation that can enter cells in the CNS independent of MCT8 include DITPA (3,5-di-iodothyropropionic acid, also known as SRW101) (45, 46) and triac (3,3′,5-tri-iodothyroacetic acid, also known as tiratricol) (19).

### DITPA

DITPA (diiodothyropropionic acid) is a synthetic T3 analog that can enter the CNS independent of MCT8 and stimulate thyroid hormone action by binding to the thyroid hormone receptors, TRα and TRβ (45, 47). Outside the brain, DITPA reduces circulating T3 by decreasing the activity of deiodinase type 1 (DIO1), which converts T4 to T3 (45). Mouse studies have shown that DITPA can reduce serum T3 levels and elicit some neurological effects, such as normalized myelination and cerebellar development, if it is administered soon after birth (during the first three post-natal weeks) (48, 49). However, despite its ability to cross the blood-brain barrier, *in vitro* studies have shown that DITPA binds to the TRβ thyroid hormone receptors with a 350-fold weaker affinity than T3 (47). In a study of four children given compassionate-use DITPA, serum T3 and TSH were reduced to normal levels, but the effect of long-term treatment has not been reported (47). DITPA is currently undergoing further study, including a clinical trial investigating its administration during pregnancy (50). DITPA (SRW101) has received FDA Orphan Drug Designation and Rare Pediatric Disease Designation (45).

### Tiratricol (triac)

Tiratricol (tri-iodothyroacetic acid) is a naturally occurring analog of T3 which can enter the brain via thyroid hormone transporters other than MCT8 (51). *In vitro* studies have demonstrated that the absence of an amino (NH2) group in the structure of tiratricol compared with T3 is associated with MCT8-independent uptake in human neuronal cells (20, 29). In addition, although tiratricol and T3 bind to the TRα1 thyroid hormone receptor with equal affinity, tiratricol binds thyroid hormone receptors TRβ1 and TRβ2 with an almost 10-fold higher affinity than T3, indicating that tiratricol can not only enter cells where T3 cannot, but it may also elicit a biological response at lower cellular concentrations (52).

Preclinical knockout mouse models have shown that tiratricol can restore neural differentiation, improve white matter loss, and promote dendritic growth and myelination in T3-depleted cells when administered soon after birth (during the first three post-natal weeks), showing promise in potentially mitigating the neurological effects of MCT8 deficiency (29, 30, 52). In another study, high doses of tiratricol were administered by intracerebroventricular delivery in three-month-old knockout mice (53). Here, the treatment could normalize the endocrinological effects of thyrotoxicosis but, despite wide cerebral tiratricol distribution, the treatment had minimal thyromimetic activity in the brain (53). This may be due to the non-neonate mice used in the study. Research comparing DITPA and tiratricol in newborn knockout mice indicated that tiratricol exerted a stronger thyromimetic effect than DITPA in promoting CNS maturation and function (49).

Several clinical trials have been completed, or are underway, to evaluate the use of tiratricol in individuals with MCT8 deficiency. The Triac I trial was an international, single-arm, open-label, phase II trial in which forty-five patients [median baseline age: 7.1 years (range 0.8–66.8)] were treated with oral tiratricol and had at least one follow-up measurement of thyroid function (19). Forty patients completed the 12-month trial (19). All the study's primary endpoints were met, including the lowering of serum total T3, with associated improvements in body weight for age, blood pressure, and resting heart rate and rhythm, with minor improvements in motor function for pediatric patients (19).

A subsequent follow-up retrospective cohort study was completed to evaluate the long-term efficacy of tiratricol on clinical and biochemical parameters (54). Patients from the Triac I trial (*n* = 27), together with individuals from a tiratricol compassionate use program (*n* = 40), were enrolled [median baseline age: 4.6 years (range 0.5–66.0)] (54). The decrease in mean serum T3, serum TSH, and free and total serum T4 was maintained in all patients with 90% (60/67) of patients achieving the primary endpoint of maintaining T3 concentrations within the target range (54). In patients treated for more than 2 years, serum thyroid hormone levels after 1 year of treatment did not differ significantly compared to their last visit and remained significantly reduced compared to baseline. This indicated a sustained benefit from tiratricol, an effect seen in patients of different ages (54).

A real-world study of four patients assessed at Hospital J. P. Garrahan in Argentina between January 2019 and December 2022 [median age at diagnosis: 1.38 years (range 0.58–7.16)]; median time from diagnosis to tiratricol initiation: 2.7 months [±2.2 months]) found serum T3 levels decreased with treatment in all four patients (55). Muscle tone and developmental delay also improved, and two children with malnutrition gained weight, highlighting that timely diagnosis and therapeutic intervention are associated with positive clinical effects (55). In a recent case report, early intervention with tiratricol in a boy diagnosed with MCT8 deficiency at twenty-one months found that, after a year of treatment, there were significant improvements in both neuronal and endocrinological symptoms, with improvement in head control, increase in body weight, and normal heart rate and blood pressure all recorded (56).

Two additional clinical trials are ongoing: the Triac II trial (57) and ReTRIACt (58). Triac II is an open-label, phase II trial, which enrolled twenty-two MCT8 patients ≤30 months of age at the onset of treatment with tiratricol. The study aims to confirm the effect of tiratricol on the biochemical changes giving rise to the endocrinological symptoms of MCT8 deficiency seen in the Triac I trial as well as investigating the potential positive impact on neurodevelopment from initiation of tiratricol treatment earlier in life (54). The ninety-six-week results are currently being analyzed and there is an ongoing two-year extension of treatment with assessment at years three and four underway (57).

The ReTRIACt trial launched recruitment in 2023 to enroll patients in a double-blind, randomized, phase III, multicenter, placebo-controlled study in approximately sixteen patients (≥4 years of age) (58). The trial aims to confirm that tiratricol administration is responsible for reducing circulating serum T3 levels (58). Tiratricol has an Orphan Drug Designation for MCT8 deficiency and resistance to thyroid hormone type beta (RTH-β) in the US and the European Union (EU) (59).

### Novel preclinical model systems

In addition to mouse models, the effect of DITPA and tiratricol have been investigated in cerebral organoids generated from induced pluripotent stem cells from MCT8-deficient patients, providing a species-specific preclinical model (60). These cells exhibited altered early neurogenesis, impaired T3 transport, and altered gene expression of thyroid hormone transporters. Administration of DITPA and tiratricol to these cerebral organoids triggered normal activity in T3-responsive genes, providing evidence that MCT8 is critical for early neurogenesis and that both treatments can elicit thyroid hormone signaling responses in human MCT8-deficient neural cells (60).

### Gene therapy

Gene therapy is being investigated to recover MCT8 function by using viral expression vectors carrying the functional *SLC16A2* gene and relevant promoter sequences to deliver wild-type MCT8 protein to MCT8-deficient cells (7). A preliminary preclinical study examined whether the transfer of human MCT8 (hMCT8) cDNA in an adeno-associated virus 9 (AAV9) vector could rescue neurological defects in Mct8 knockout mice. Intravenous (IV) AAV9-hMCT8 increased the activity of MCT8, with a significant increase in brain T3 content (61). More recent gene therapy studies in mice have demonstrated the long-term benefits of IV AAV9-hMCT8 and IV AAV-BR1-Mct8 on the CNS of mice, with both increasing brain T3 content (62, 63).

### Chemical chaperones

Chemical chaperones are also being investigated in the setting of MCT8 deficiency. Preliminary preclinical data have demonstrated that sodium phenylbutyrate (NaPB) can stabilize the protein-folding defects of certain pathogenic *SLC16A2* mutations, increase MCT8 expression in the cell membrane, and improve T3 transport in cells with various *SLC16A2* mutations (2, 64). Although chaperones like NaPB show promise, it is likely they will only work in a subset of patients with *SLC16A2* mutations associated with misfolding and increased protein degradation (64). Another potential treatment option is the CNS-selective amide prodrug sobetirome (Sob-AM2) (65). Maternal administration of Sob-AM2 can cross the placenta and access fetal tissues, including the brain, in the absence of MCT8, modulating the expression of T3-dependent genes (65). However, in preclinical mouse trials, sobetirome treatment led to spontaneous abortions. Further investigations are needed to determine how this type of treatment could potentially help prevent neurodevelopmental alterations in the MCT8-deficient fetal brain with reduced toxicity (65).

## Summary

The results of this narrative review highlight that MCT8 deficiency is a chronic condition for which there are several areas of unmet need, with resultant delays in diagnosis and a shortfall in access to multidisciplinary management. Parents and caregivers continue to struggle with the complex patient journey, which is compounded by the lack of an effective, approved therapy, as well as the small window where treatment may have the greatest impact on neurodevelopmental outcomes.

While there is evidence of increased awareness of MCT8 deficiency among HCPs, reflected by a decreased time to diagnosis, there remains a need for improved early recognition and therapeutic interventions that will have the most significant clinical impact if initiated in pregnancy or as early in the patient's life as possible (4). The understanding of this disorder is improving with the establishment of patient registries and international rare disease networks which provide vital caregiver information on the natural history of a disorder to help supplement information gathered from clinical trials (4, 12, 66, 67). Continued education of HCPs from different specialties is needed to ensure MCT8 deficiency is detected at the earliest opportunity and patients are not passed from specialist to specialist to obtain a diagnosis.

An early diagnosis of MCT8 deficiency is essential to providing the patient with supportive care which can greatly enhance the quality of life of patients and caregivers (6). It is important that patients receive the appropriate complement of thyroid hormone tests to aid a diagnosis of MCT8 deficiency. The diagnostic thyroid hormone “fingerprint” profile includes elevated serum T3 levels with low T4 and normal to mildly elevated TSH results. In the neonate, T3 is not elevated; however, rT3 is low (26, 68). Earlier diagnosis of the disorder in the future could allow for more prompt intervention with newer therapies and the potential clinical benefits those provide.

As MCT8 deficiency is an inherited condition, carrier testing of at-risk female relatives, prenatal testing for a pregnancy at increased risk, and preimplantation genetic testing are possible, but this is only practicable in families where an affected child has already been born and there are the financial means to pursue this approach (1, 4, 26). Preclinical studies have highlighted the potential of prenatal molecular chaperone treatment (65), and a trial is underway to evaluate DITPA treatment of affected children *in utero* to determine if treating MCT8 deficiency before birth has a positive impact on fetal neurodevelopment (51).

If individuals with suspected MCT8 deficiency are evaluated by whole genome or whole exome sequencing, it is important to ensure that the *SLC16A2* gene is a locus that is analyzed and reported. Similarly, panels developed to screen for developmental delay or thyroid hormone-related disorders should include *SLC16A2* so a diagnosis of MCT8 deficiency is possible. As access to genetic testing improves, there is an increased understanding of the genotype-phenotype relationships in MCT8 deficiency, as well as the variance in the degree of clinical expression of the disorder (6, 12, 21). To help with further identification of cases, data should be regularly reanalyzed, and databases reinterrogated to ensure up-to-date categorization of any variants found (43).

With affected individuals largely dependent on caregivers throughout their lives (4), it's vital to understand what these families need to improve their quality of life. Capturing patient-reported outcomes can help meet the specific needs of each family and their preferred therapeutic goals (4, 12, 69). The needs of patients with complex disorders are dynamic and change over time, and their supportive care team should adapt and respond as the child grows into adulthood (69, 70). The International Rare Diseases Research Consortium (IRDiRC) task force is committed to developing patient-relevant outcomes, stating patients and/or their representatives should be involved in all relevant aspects of research, an essential step in the development of more patient-focused research outcomes (71).

There remains a significant unmet need for new treatments specifically evaluated in, and approved for, MCT8 deficiency. To effectively and specifically treat MCT8 deficiency, a therapeutic molecule must be able to enter the brain independent of the MCT8 protein; have a high affinity for thyroid hormone receptors; be able to elicit T3-like effects on genes downstream of the thyroid hormone receptors; preserve or restore neural differentiation in the brain; alleviate endocrinological symptoms due to MCT8 deficiency, and demonstrate good long-term safety and tolerability (16, 19, 29, 44). Tiratricol is furthest along its developmental pathway as a targeted treatment for MCT8 deficiency. In October 2023, an EU marketing authorization application (MAA) for tiratricol (Emcitate®) in the treatment of MCT8 deficiency was submitted and an application to the US FDA is anticipated soon (72).

There are multiple challenges in the diagnosis and management of MCT8 deficiency. To improve patient outcomes, MCT8 deficiency awareness must be increased across the clinical and scientific communities involved in diagnosing and supporting these patients, including general practitioners, pediatric neurology, pediatric genetics, endocrinology, gastroenterology, nutritionists and dieticians, nurse specialists, and supportive care therapists. A definitive diagnosis can empower families and lead to clinical, practical, emotional, and financial support. Earlier intervention and emerging treatments offer hope for improved prognosis and better patient and caregiver quality of life.
